# Heterogeneous persistence of *Mycobacterium leprae* in oral and nasal mucosa of multibacillary patients during multidrug therapy

**DOI:** 10.1590/0074-02760220058

**Published:** 2022-10-17

**Authors:** Arthur da Silva Neumann, Amanda Nogueira Brum Fontes, Márcia Quinhones Pires Lopes, Philip Noel Suffys, Milton Ozório Moraes, Flávio Alves Lara

**Affiliations:** 1 Fundação Oswaldo Cruz-Fiocruz, Instituto Oswaldo Cruz, Laboratório de Microbiologia Celular, Rio de Janeiro, RJ, Brasil; 2 Universidade Federal do Rio de Janeiro, Instituto de Ciências Biomédicas, Laboratório de Investigação em Neuroprogramação, Rio de Janeiro, RJ, Brasil; 3 Ministério da Saúde, Secretaria de Ciência, Tecnologia e Insumos Estratégicos, Departamento de Ciência e Tecnologia, Brasília, DF, Brasil; 4 Fundação Oswaldo Cruz-Fiocruz, Laboratório de Biologia Molecular Aplicada à Micobactérias, Rio de Janeiro, RJ, Brasil; 5 Fundação Oswaldo Cruz-Fiocruz, Instituto Oswaldo Cruz, Laboratório de Hanseníase, Rio de Janeiro, RJ, Brasil

**Keywords:** leprosy, nasal scrapings, oral scrapings, molecular viability, qPCR, treatment

## Abstract

**BACKGROUND:**

Leprosy is curable by multidrug therapy (MDT) treatment regimen ranging from six to 12 months. The variable levels of tolerance and adherence among patients can, however, result in treatment failure and the emergence of drug-resistant strains.

**OBJECTIVES:**

Describe the impact of MDT over *Mycobacterium leprae* viability in patient’s oral and nasal mucosa along treatment.

**METHODS:**

*Mycobacterium leprae* viability was monitored by quantitative polymerase chain reaction (qPCR) quantification of 16S rRNA in lateral and contralateral scrapings of oral and nasal mucosa of 10 multibacillary patients along the initial five months of treatment.

**FINDINGS:**

The results demonstrated high heterogenicity of *M. leprae* viability among patients and between nasal and oral samples. Of six patients who presented good adherence and tolerance to the treatment, only four displayed absence of *M. leprae* viability in both samples three months after the first MDT dose, while for the other two, the absence of *M. leprae* viability in the oral and nasal cavities was only detected five months after the first dose.

**MAIN CONCLUSIONS:**

We concluded that qPCR of 16S rRNA for the determination of *M. leprae* viability in nasal and oral scraping samples could represent an interesting approach to monitor treatment efficacy.

Leprosy persists as a relevant disease in tropical countries, with 202,256 new cases registered worldwide in 2019.^1^ Treatment involves a multidrug therapy (MDT) regimen consisting of a combination of dapsone, clofazimine, and rifampicin. Dapsone can be substituted for ofloxacin in some patients that experience the adverse effects of dapsone.^2^ Treatment duration can take 12 months for multibacillary patients, or even longer if an interruption to the regimen occurs. Due to the toxicity of the treatment, it has a variable level of adherence among patients, which can result in therapeutic failure as well as the emergence of drug-resistant *Mycobacterium leprae* strains.

MDT was implemented worldwide in the 1990’s with a huge success in reducing the prevalence of the disease by half within just ten years, from 10-12 million cases in 1981 to 5.5 million in 1991.^3^ Unfortunately, this rate of decrease started to slow down in 2006, stabilising at around 0.2 new cases per 10,000 inhabitants, with about 200,000 new cases reported every year worldwide.^1^ The consistent number of new cases annually highlights the need for better tools to identify the risks of transmission and monitor treatment efficacy in order to improve disease control.

Nasal secretions from untreated multibacillary patients were identified decades ago as the major factor in the spread of leprosy as they release large amounts of live bacilli into the environment.^4^ The airborne route has also been demonstrated as a potential source of *M. leprae* infection in experimental mouse models.^5^ Thus, identifying and effectively treating multibacillary patients has been the main strategy for controlling the disease. Furthermore, contact tracing of leprosy patients in order to achieve early diagnosis or initiate chemo- or immunoprophylaxis is also an effective way to control the spread of leprosy and leads to reduced disease burden.^6^ ^,^ ^7^ However, it is still unclear what risks leprosy patients undergoing MDT pose to potential contacts in terms of the spread of live bacilli, as well as the correlation between bacterial viability and the effectiveness of the treatment.

The bacilloscopic index (BI), determined by counting the acid-fast bacilli after kinyoun staining of slit-skin smear samples, even though widely used, is not a good parameter for monitoring treatment efficiency. The BI is not directly related with *M. leprae* viability and decreases very slowly, in some cases the BI remains stable for months or even years after the end of treatment.^8^

In the absence of a serologic diagnostic test able to detect paucibacillary patients, the polymerase chain reaction (PCR) technique has been used to support the diagnosis of leprosy for over a decade,^8^ using samples of skin, urine, nasal or oral swabs, eye lesions, blood, and nerves of paucibacillary and multibacillary patients.^9^ ^-^ ^16^ Among the types of samples studied, nasal swabs have shown the most promise for this purpose due to the minimal invasiveness and high bacterial load typically found in the nose.

In 2009, Martinez and collaborators developed a molecular method capable of measuring *M. leprae* viability by quantitative PCR (qPCR), normalising copy number of 16S ribosomal RNA (rRNA) transcripts against 16S rRNA DNA.^8^ The method is based on the inherent instability of the RNA molecule, used as a normaliser of viability, and the innate stability of the DNA molecule, used as a normaliser of bacillary load. Using this approach, the authors successfully demonstrated the feasibility to detect *M. leprae* viability in skin biopsies before and after treatment, showing in some cases persistence of genetic material of the pathogen years after treatment.^11^ The qPCR technique is also associated with high sensitivity, with a detection limit of 20 fg of *M. leprae* DNA, equivalent to four bacilli.^17^

In this study, for the first time, we analysed *M. leprae* viability by qPCR of the 16S rRNA in samples taken from the oral and nasal cavities of a selection of multibacillary patients during the first five months of MDT. In two patients, we observed *M. leprae* viability in the nasal cavities until the fourth month of treatment, which was apparently not due to drug resistance (as verified by Sanger sequencing of drug resistance-associated genes). The influence of other factors that could impact bacillus viability, such as treatment intolerance and subsequent interruption and reactional episodes, are also discussed.

## SUBJECTS AND METHODS

*Patients and samples* - Ten multibacillary leprosy outpatients (four females and six males; median age: 40 years; age range: 12-62 years) with BI above 3.5 were recruited from the Fiocruz Leprosy Clinic (Rio de Janeiro, Brazil) (Table). Leprosy was diagnosed by clinical, bacteriological, and histological criteria. After signing the Informed Consent Form approved by the Human Research Ethics Committee (No. 1950.165), monthly scrapings (just before and during the first five months of MDT) of both nasal cavities and cheeks using a sterile plastic scraper were collected. All individuals were monitored annually at the Fiocruz Leprosy Clinic after clinical cure and all individuals were considered healthy after five years. All patients presented reactional episodes after cure except for patient 6.

**TABLE:** Clinical profile of leprosy patients

Patient	Sex	Age	BI at admission	Leprosy cases / investigated household contacts^*^	Treatment (months)	Intercurrence	BI after 12 months of treatment	Reactional episode after cure
1	M	38	5	0/4	R/C/D (0-12)	No	1.75	Yes
2	M	44	5	0/3	R/C/D (0-12)	No	3.75	Yes
3	F	56	5.5	0/11	R/C/D (0-12)	No	4.75	Yes
4	F	40	5.75	1/2	R/C/D (0-12)	No	4.75	Yes
5	M	62	4	0/3	NT (0-4) R/C/O (4-12)	Pneumonia / Hemolytic anaemia	4	Yes
6	M	41	4	0/4	R/C/D (0-12)	No	2.5	No
7	F	12	5	2/4	R/C/D (0-12)	No	4.75	Yes
8	M	54	4.75	0/11	R/C/O (0-12)	No	2.3	Yes
9	F	20	5	1/20	R/C/D (0-2) NT (2-4) R/C/O (4-12)	Dapsone sensitivity / Hemolytic anaemia	4.85	Yes
10	M	33	5.25	1/11	R/C/D (0-12)	No	3.7	Yes

M: male; F: female; BI: bacilloscopic index; R/C/D: rifampicin, clofazimine, and dapsone (conventional treatment); R/C/O: rifampicin, clofazimine, and ofloxacine (alternative treatment); NT: not treated; *all household contacts leprosy cases were diagnosed as multibacillary.

*Mycobacterium leprae viability quantification in oral and nasal scrapings* - The viability of *M. leprae* was determined after simultaneously extraction of RNA and DNA from each sample by TRIzol^TM^ reagent (Invitrogen) as described elsewere.^16^ Briefly, 500 µl of TRIzol™ Reagent (Invitrogen) was immediately added to the collected samples, which were then homogenised by vortexing, incubated for 30 min at room temperature cooled for 5 minutes and then 100 µL of chloroform was added. After rapid mixing by inversion, the tubes were centrifuged at 1200 x g at 4**º**C for 15 min. The supernatant aqueous layer containing RNA was transferred to a new tube and precipitated with isoamyl alcohol. The RNA pellet was centrifuged for 30 min at 12,000 x g and washed once with ethanol 70%. The pellet was dissolved in 30 µL of sterile distilled water and stored at -70**º**C until use. DNA was purified from the organic phase. 50 µL of 10mM Tris-EDTA solution (pH 8.0) and 75 µL of chloroform-isoamyl alcohol solution (24:1) were added and homogenised by vortexing. After centrifugation at 12,000 x g for 10 min at 4**º**C, the aqueous phase was transferred to another tube and the DNA was precipitated with isoamyl alcohol. The DNA pellet was washed in 70% ethanol, centrifuged for 10 min at 10,000 x g, dissolved in 20 µL of sterile distilled water, and stored at -70**º**C until use.

For the removal of DNA to obtain RNA, samples were treated using the TURBO DNA-free kit (Thermo Fisher Scientific), following the manufacturer’s recommendations. The quantification of RNA and DNA samples was performed using the NanoDrop™ 1000 spectrophotometer (Thermo Fisher Scientific). RNA (500 ng) was then used for cDNA synthesis using the GoScript™ Reverse Transcription System kit (Promega), following the manufacturer’s recommendations. Levels of *M. leprae* 16S rRNA were determined by real-time PCR (qPCR) using 50 ng of DNA and cDNA. Reactions were performed using TaqMan^®^ PCR Universal Master Mix (Thermo Fisher Scientific), TaqMan^®^ probe and 16S rRNA Primer Mix (Supplementary data - Table), using a StepOne Plus^®^ time PCR system (Applied Biosystems). As a control for DNA contamination of the cDNA, the DNase-treated RNA samples were used in the qPCR reactions in the absence of reverse transcriptase. Additionally, negative (water) and positive controls (purified *M. leprae* DNA) were added to each experiment. The *M. leprae* viability was expressed by a twofold Cts value curve (2-ΔΔCT), measuring the levels of 16S rRNA cDNA, and normalising against the bacillus DNA.

Analysis was performed in duplicate using the lateral and contralateral of the oral and nasal samples and normalised in the GraphPad Prism 5 program by assigning 100% of viable *M. leprae* load to the highest value of each temporal analysis. Absence of 16S rRNA transcript detection was determined as 0%. The non-parametric Friedman test was performed by comparing each time point with the next one.

*Amplification and sequencing analysis of part of rpoB, folP1, and gyrA* - About 200 ng of nasal DNA samples collected at each time point were submitted to PCR using primers targeting three main *M. leprae* genes related to resistance: *fol*P1, *gyr*A, and *rpo*B (Supplementary data - Table) according to Avanzi and collaborators.^18^ Briefly, DNA was denatured at 94ºC for 15 min, and then amplified by PCR in a Veriti™ 96-Well Thermal Cycler (Applied Biosystems) by submitting samples to six cycles consisting of 94ºC for 45 s, 68ºC for 45 s, 63ºC for 45 s, and 72ºC for 90 s followed by 35 cycles of 94ºC for 45 s, 62ºC for 45 s, and 72ºC for 90 s, with a final extension at 72ºC for 10 min. For evaluation of PCR yield, gel electrophoresis was performed using the PCR product, and amplicons were purified using the QIAquick PCR Purification kit (QIAGEN), followed by sequencing using the ABI PRISM BigDye™ Terminator v 3.0 Sequencing kit (Applied Biosystems) using 30 cycles of 96ºC for 45 s, 50ºC for 5 s, and 60ºC for 10 min. Analysis was performed using the ABI Prism 3730 Automated DNA Sequencer (Applied Biosystems) and the Molecular Evolutionary Genetics Analysis (MEGA) version 7.0 program.

## RESULTS

Information on the clinical and bacteriological characteristics, as well as the treatment schemes, of the patients enrolled in the present study are given in Table. *M. leprae* viability curves were generated by the RNA/DNA ratio for the *M. leprae* 16S rRNA gene, as determined by qPCR, from the oral and nasal scrapings of the patients (Figure). Curiously, we observed that, throughout the treatment period, there were peaks of *M. leprae* viability in both samples. Some of these viability peaks could be explained by the interruption of treatment, represented as a broken line, and indicated in the x-axis of the graphs of patients 5, 8, and 9. This, however, cannot explain the increases seen in the samples of patients 1, 7, and 10, as these patients presented good tolerance to the treatment and received a monthly assisted dose of rifampicin.

**Figure:**
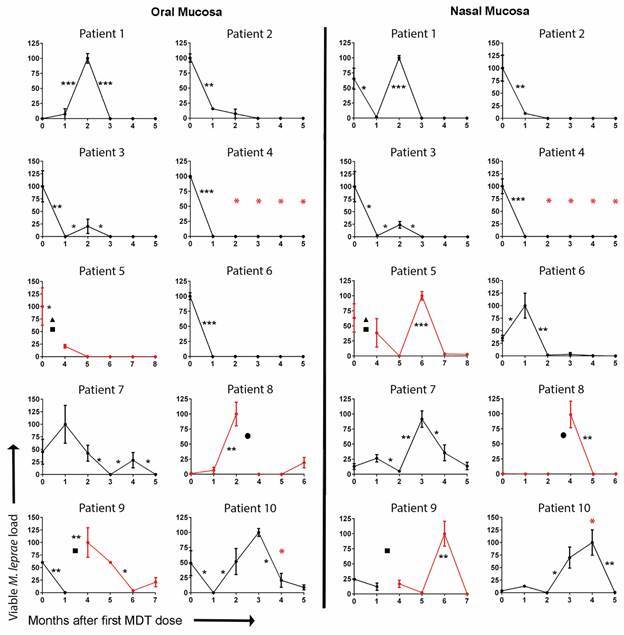
*Mycobacterium leprae* molecular viability analysis in oral and nasal scrapings from multibacillary patients. *M. leprae* molecular viability over the first five months of the multidrug therapy (MDT) regime. For each patient’s temporal series (oral or nasal), the highest viability observed was arbitrary determined as 100% of viable *M. leprae* load, while 0% indicates the absence of 16S rRNA or absence of viable *M. leprae*. The black line represents conventional MDT (rifampicin, clofazimine, and dapsone); the red line represents alternative MDT (rifampicin, clofazimine, and ofloxacin); the broken line represents interruption of treatment. The red asterisk indicates erythema nodosum leprosum occurrence; the triangle indicates pneumonia; the square indicates dapsone-associated hemolytic anaemia; the circle indicates treatment-related fever and asthenia. The non-parametric Friedman test was performed by comparing each time point with the next one, where * means p value < 0.05, ** means p < 0.005, and *** means p < 0.001.

All patients presented an absence of the 16S rRNA signal in at least one of the samples in the time interval between one and five months after the first treatment dose. It is important to note that even though patients showed a drastic reduction in the viability of the bacillus in their oral mucosa after the first dose of MDT (patients 2, 3, 4, 5, and 6), some demonstrated signs of viable *M. leprae* in their nasal mucosa up to the second month after the first dose (patients 5 and 6). Considering all the patients who did not have their treatments interrupted, the time to achieve absence of *M. leprae* viability in both oral and nasal cavities ranged from two to five months after the first dose, except for patient 4, who already displayed an absence of *M. leprae* viability at 30 days after the first dose of MDT. This time range is extended as patients 7 and 10 curiously showed a viability peak of the bacillus in the nasal cavity samples three and four months, respectively, after the administration of the first dose. The reinfection of these patients’ nasal cavities along treatment by infected household contacts could not be discarded, since two multibacillary leprosy patients were identified in the household contacts of patient 7, one related with patient 9 and one related with patient 10 (Table).The considerable differences observed between the *M. leprae* viability profiles of the patients spurred the investigation into the potential drug resistance of the *M. leprae* in the samples by sequencing specific regions of the *fol*P1, *gyr*A, and *rpo*B genes. We could not detect any antibiotic resistance polymorphisms (data not shown), which was corroborated by the clinical aspects of these patients at the end of the treatment, as they were all considered cured.

This cohort captured the current situation related to the treatment of leprosy. Among the 10 patients followed up in this study, three had complications related to MDT adverse effects: two suffered from dapsone-associated hemolytic anaemia (patients 5 and 9) and another presented treatment-related fever and asthenia (patient 8). The conventional MDT had to be discontinued in these three patients and we observed that this led to an increase in the *M. leprae* viability in the oral sample of patient 9 and nasal samples of all three patients. Upon resuming treatment with the replacement of dapsone by ofloxacin, a drastic reduction in the viability of *M. leprae* was observed after the first dose (patient 8) or after the third dose (patients 5 and 9).

It is interesting to note that viability increments in the oral scaping, related or not to interruption of treatment, were accompanied by similar viability increments in the nasal scraping, generally with a delay of two months, as observed in patients 7, 8, and 9, or one month in the case of patients 6 and 10. We could also observe the occurrence of two cases of erythema nodosum leprosum during the first months of treatment (patients 4 and 10), apparently without interference in *M. leprae* viability.

## DISCUSSION

Despite the general belief by physicians and health professionals that multibacillary patients stop being a relevant source of infective *M. leprae* after the administration of the first dose of MDT,^19^ there is a lack of studies on the viability of *M. leprae* in the oral and nasal cavities of these patients throughout the treatment.

The classic Shepard’s method to monitor *M. leprae* viability, efficacy of treatment, and drug resistance is based on bacterial growth in the mouse footpad and is a labor-intensive and time-consuming procedure.^20^ In the present work, we apply qPCR of the 16S rRNA gene to monitor *M. leprae* viability in clinical samples from 10 patients during the first five months of treatment.

We observed that, on the contrary to the common belief, even patients that took the rifampicin dose with the correct periodicity still presented viable *M. leprae* in their nose and/or mouth from two to four months after the first dose. This data is in accordance with those of Davis and colleagues, who observed a reduction in *M. leprae* viability only after the administration of five doses of rifampicin in athymic nu/nu mice.^21^

None of the *M. leprae* strains from this cohort of patients presented a high confidence mutation associated with antibiotic resistance in the *fol*P1, *gyr*A, or *rpo*B genes. In accordance with that, none of the patients presented a relapse after five years of follow-up. For this reason, we assume that these variations in *M. leprae* viability throughout treatment are related to factors other than these known resistance mechanisms of the bacillus to MDT. Such mechanisms involved could be host immunity, the patient’s drug metabolisation profile, genetic background, and/or adherence to treatment, since only the administration of rifampicin was supervised.

Several studies that were based on nucleic acid amplification have demonstrated that nasal cavities are an important reservoir of *M. leprae* in humans.^22^ ^,^ ^23^ ^,^ ^24^ ^,^ ^25^ ^,^ ^26^ Using a similar approach, researchers detected *M. leprae* viability from the nasal swabs of 50% (10 out of 20) of non-treated multibacillary patients.^26^ In our cohort of patients, we observed that 30% (3 out of 10) of the patient nasal scrapings were negative before treatment (patients 6, 8, and 10). Curiously, all three patients became positive during the treatment period before returning to a negative state again. This indicates that *M. leprae* viability assessment of the nasal mucosa is a dynamic event, and for that reason PCR assessment of a nasal swab sample taken at a single time point might not be a useful tool in *M. leprae* diagnosis and/or control.

Another limitation of this tool is the persistence of *M. leprae* viability in the oral or nasal mucosa due to continuous exposition during treatment to infected household contacts, as observed in patients 7, 9 and 10. On the other hand patient 4, which presented a strong and persistent drop in *M. leprae* viability, in both oral and nasal mucosa, also presented one multibacillary new case among household contacts.

Our findings also demonstrate a certain correlation of viability profiles in the nose and mouth cavities. Although four patients presented a similar *M. leprae* viability level in the mouth and nose environment (patients 1, 2, 3, and 4), another four presented a higher persistence of *M. leprae* viability in the nose. Since the method used to determined *M. leprae* viability is based on a relation between RNA and DNA, and therefore not strictly related to the number of *M. leprae* bacilli present, this difference observed between the oral and nasal samples cannot be explained by differences in bacterial load or presence of PCR inhibitors in oral samples. However, earlier studies have demonstrated that many other environmental factors could impact the detection on *M. leprae* DNA in nasal swabs, such as air humidity.^27^

We observed considerable heterogeneity in the quantity of *M. leprae* RNA in the mouth and the nose of this cohort of patients ranging from 30 days to five months after the first dose of MDT. We also observed a substantial increase in *M. leprae* viability after interruption of treatment, followed by a significant drop after implementation of and adherence to an alternative treatment.

In conclusion, monitoring of *M. leprae* viability by 16S rRNA qPCR in nasal and oral scraping samples throughout treatment seems to be a promising tool to monitor treatment efficacy and could be useful for transmission surveillance among patient contacts and in the general population in epidemiological studies.
