# Supplementary material for: Heterogeneous persistence of Mycobacterium leprae in oral and nasal mucosa of multibacillary patients during multidrug therapy
Source: Mem Inst Oswaldo Cruz. 2022 Oct 17;117:e220058. doi: 10.1590/0074-02760220058 (PMC9575966; doi:10.1590/0074-02760220058)
Supplement: Supplementary file 1 [file 1678-8060-mioc-117-e220058-s.pdf]

TABLE

Target gene/probes	Primers	Amplicon size	Reference
Probe 16S	6FAM - CATCCTGCACCGCA – MGBNFQ	-	(8)
16S rRNA	Forward 5’-GCATGTCTTGTGGTGGAAAGC-3’	71 bp	
	Reverse5’-CACCCCACCAACAAGCTGAT-3’		
*fol*P1	Forward 5’-TACTTACTGTAATCCCCTGTGCTG-3’	173 bp	(28)
	Reverse 5’-TTGATCCTGACGATGCTGTC-3’		
*rpo*B	Forward 5’-GGTGGTCGCCGCTATCAAG-3’	289 bp	
	Reverse 5’-TTTGCGGTACGGTGTTTCG-3’		
*gyr*A	Forward 5’-CCCGGACCGTAGCCACGCTAAGTC-3’	178 bp	
	Reverse 5’-CATCGCTGCCGGTGGGTCATTA-3’		

CC BY
